# Analysis and comparisons of gene expression changes in patient- derived neurons from ROHHAD, CCHS, and PWS

**DOI:** 10.3389/fped.2023.1090084

**Published:** 2023-05-10

**Authors:** A. Kaitlyn Victor, Tayler Hedgecock, Martin Donaldson, Daniel Johnson, Casey M. Rand, Debra E. Weese-Mayer, Lawrence T. Reiter

**Affiliations:** ^1^ IPBS Program, Neuroscience Institute, University of Tennessee Health Science Center, Memphis, TN, United States; ^2^ Department of Neurology, University of Tennessee Health Science Center, Memphis, TN, United States; ^3^ Department of Pediatric Dentistry and Community Oral Health, University of Tennessee Health Science Center, Memphis, TN, United States; ^4^ Molecular Bioinformatics Core, University of Tennessee Health Science Center, Memphis, TN, United States; ^5^ Department of Pediatrics, Division of Autonomic Medicine, Ann & Robert H. Lurie Children’s Hospital of Chicago and Stanley Manne Children’s Research Institute, Chicago, IL, United States; ^6^ Department of Pediatrics, Northwestern University Feinberg School of Medicine, Chicago, IL, United States; ^7^ Department of Pediatrics, University of Tennessee Health Science Center, Memphis, TN, United States; ^8^ Department of Anatomy and Neurobiology, University of Tennessee Health Science Center, Memphis, TN, United States

**Keywords:** autonomic dysfunction, neurogenetic syndromes, dental pulp stem cells, mRNA seq, genomics

## Abstract

**Background:**

Rapid-onset obesity with hypothalamic dysfunction, hypoventilation, and autonomic dysregulation (ROHHAD) syndrome is an ultra-rare neurocristopathy with no known genetic or environmental etiology. Rapid-onset obesity over a 3–12 month period with onset between ages 1.5–7 years of age is followed by an unfolding constellation of symptoms including severe hypoventilation that can lead to cardiorespiratory arrest in previously healthy children if not identified early and intervention provided. Congenital Central Hypoventilation syndrome (CCHS) and Prader-Willi syndrome (PWS) have overlapping clinical features with ROHHAD and known genetic etiologies. Here we compare patient neurons from three pediatric syndromes (ROHHAD, CCHS, and PWS) and neurotypical control subjects to identify molecular overlap that may explain the clinical similarities.

**Methods:**

Dental pulp stem cells (DPSC) from neurotypical control, ROHHAD, and CCHS subjects were differentiated into neuronal cultures for RNA sequencing (RNAseq). Differential expression analysis identified transcripts variably regulated in ROHHAD and CCHS vs. neurotypical control neurons. In addition, we used previously published PWS transcript data to compare both groups to PWS patient-derived DPSC neurons. Enrichment analysis was performed on RNAseq data and downstream protein expression analysis was performed using immunoblotting.

**Results:**

We identified three transcripts differentially regulated in all three syndromes vs. neurotypical control subjects. Gene ontology analysis on the ROHHAD dataset revealed enrichments in several molecular pathways that may contribute to disease pathology. Importantly, we found 58 transcripts differentially expressed in both ROHHAD and CCHS patient neurons vs. control neurons. Finally, we validated transcript level changes in expression of *ADORA2A*, a gene encoding for an adenosine receptor, at the protein level in CCHS neurons and found variable, although significant, changes in ROHHAD neurons.

**Conclusions:**

The molecular overlap between CCHS and ROHHAD neurons suggests that the clinical phenotypes in these syndromes likely arise from or affect similar transcriptional pathways. Further, gene ontology analysis identified enrichments in ATPase transmembrane transporters, acetylglucosaminyltransferases, and phagocytic vesicle membrane proteins that may contribute to the ROHHAD phenotype. Finally, our data imply that the rapid-onset obesity seen in both ROHHAD and PWS likely arise from different molecular mechanisms. The data presented here describes important preliminary findings that warrant further validation.

## 1. Introduction

**R**apid-onset **O**besity with **H**ypothalamic dysfunction, **H**ypoventilation, and **A**utonomic **D**ysregulation (ROHHAD) is a devastating disorder that affects children primarily between the ages of 1.5–7 years old with a rapid onset of symptoms (1–3). ROHHAD is an ultra-rare neurocristopathy with only 200 cases described or identified to date (3). The ROHHAD acronym describes the typical order in which this disease unfolds. Rapid-onset obesity, 20–30 pounds over a 3–12 month period, is typically the first symptom that appears for ROHHAD patients (3). This rapid-onset obesity is followed by hypothalamic issues including altered salt/water balance, hypothyroidism, growth hormone insufficiency, altered pubertal onset, and additional hypothalamic symptoms (2). The autonomic nervous system (ANS) dysfunction in ROHHAD includes gastrointestinal (GI) dysfunction, ophthalmologic issues, thermal dysregulation, and cardiac dysrhythmia (2). Nearly half of all ROHHAD patients will present with a neural crest tumor, typically a ganglioneuroma or ganglioneuroblastoma, but rarely a neuroblasoma (3). Alveolar hypoventilation is the most devastating symptom, as it can have a stealth onset and result in cardiorespiratory arrest, potentially leading to severe morbidity or sudden death in previously healthy children (1).

Currently there is no known genetic cause for ROHHAD. The leading theories for the cause of ROHHAD are genetic, epigenetic and autoimmune based (3–6). Potentially causative genes that have roles in nervous system development have been investigated including *BDNF*, *ASCL1*, *NDN*, *ADCYAP1*, *OTP*, *PACAP*, and *HTR1A* (2, 7, 8). None of these genes could be correlated to ROHHAD symptomology. Barclay et al. sequenced the exomes of seven ROHHAD trios and found that no two subjects had the same *de novo* variants (4). Monozygotic twins discordant for ROHHAD were also sequenced with no genetic coding differences noted (4). The authors suggest the possibility of varying epigenomes between the identical female twins leading to this discordance. The presence of anti-hypothalamus and anti-pituitary antibodies in cerebrospinal fluid of ROHHAD patients suggests a possible immune system mediated pathogenesis, although only three case studies have been reported (6, 9). Additionally, immunosuppressive drugs have been found to have a positive effect on the neurological function of one ROHHAD subject (10). To date, no underlying genetic or immunologic etiology has been identified that can explain the ROHHAD phenotype.

Currently, ROHHAD is diagnosed based on clinical presentation after a related syndrome, **C**ongenital **C**entral **H**ypoventilation **S**yndrome (CCHS) is ruled out. Similar to ROHHAD, CCHS is a rare neurocristopathy with approximately 3,000 cases identified since 1970 (3). CCHS subjects typically present with hypoventilation from birth and all subjects require ventilatory support (3, 11–13). Unlike ROHHAD, CCHS is caused by a genetic lesion in *PHOX2B*, a transcription factor, primarily from polyalanine repeat expansion mutations (PARMs). The lengths of the PARM repeats correlate linearly with severity of symptoms while a subset of CCHS patients possessing non-PARMs in the *PHOX2B* gene present with more severe CCHS phenotypes (3, 11). CCHS presents with both ANS dysfunction and hypoventilation, however, there is no rapid-onset obesity and only rarely hypothalamic dysfunction. In terms of ANS dysregulation, CCHS and ROHHAD exhibit overlapping symptoms including ophthalmologic issues, GI dysmotility, and increased pain perception thresholds (11–13). Some CCHS patients, typically with an NPARM mutation in *PHOX2B*, also present with neural crest tumors. The overlapping phenotypes in these syndromes imply a similar molecular etiology, although formal molecular links between ROHHAD and CCHS have not yet been identified.

Prader-Willi syndrome (PWS) is a hypothalamic disorder with some overlapping phenotypic features to ROHHAD including the rapid-onset obesity (14). PWS is caused by the loss of paternal specific expression of the genes *SNRPN, SNURF, SNORD116* and *MAGEL2* in the PWS critical region (15q11.2–q13.3) on the 15th chromosome. Although the trajectory of the rapid-onset childhood obesity in PWS is less steep than that seen in ROHHAD, and the age at onset differs, the obesity is a defining feature of PWS and is accompanied by extreme hyperphagia (15). Autonomic and hypothalamic dysfunction are present in both PWS and ROHHAD syndrome. Hypothalamic dysfunction overlapping both syndromes includes hypothyroidism, growth hormone insufficiency, and altered pubertal onset. Autonomic dysfunction overlapping the two syndromes includes GI dysmotility, ophthalmologic manifestations, elevated pain threshold and thermal dysregulation (2, 3, 8, 15, 16). Due to these overlapping clinical features, the PWS critical region has been studied in a ROHHAD cohort. Using both next-generation sequencing and methylation sensitive assays of the paternally expressed genes in the region, no variation or loss of heterozygosity was found in the ROHHAD cohort for this region of interest (14). Although no genes within the 15q11.2–q13.3 region were found to be perturbed in ROHHAD, the similarities between these syndromes leave open the possibility that the same molecular pathways are co-regulated and warrants further gene expression studies in patient-derived neurons.

To understand the molecular overlap among ROHHAD, CCHS, and PWS, we performed RNA sequencing (RNAseq) on neurons derived from dental pulp stem cells (DPSC) collected from ROHHAD and CCHS individuals as well as neurotypical controls. DPSC are multipotent stem cells of neural crest origin and have been differentiated to a variety of cell types including chondrocytes, adipocytes, and neurons (17–19). DPSC have been found to recapitulate the epigenetic environment of embryonic stem cells more accurately than induced pluripotent stem cells and are obtainable without the need for invasive biopsies or viral reprogramming (20, 21). Many groups have differentiated DPSC to neuronal lineages showing morphological, transcriptional, and functional characteristics of terminally differentiated neurons (22–36). Our group and others have had success modeling neurogenetic syndromes using these stem cells differentiated to neuronal cultures (37–41). Using this unique patient-derived stem cell model, we have observed molecular signatures and cellular phenotypes of various syndromes in primary neurons including Prader-Willi, Angelman, and Duplication 15q syndromes (42–44). Here we utilize this system to differentiate patient-derived DPSC lines to neuronal cultures for RNAseq in order to identify the molecular similarities and find genotype/phenotype correlations among ROHHAD, CCHS and PWS. Early diagnosis and clinical intervention is critical for ROHHAD patients, so identifying molecular markers distinguishing ROHHAD from clinically related syndromes is as important as identifying the molecular commonalities between these syndromes with known genetic lesions.

## 2. Materials and methods

### 2.1. Obtaining teeth for DPSC cultures

Neurotypical control teeth were obtained through the Department of Pediatric Dentistry and Community Oral Health at the University of Tennessee Health Science Center (UTHSC). Teeth from children with ROHHAD and CCHS were collected remotely by the caregivers of these subjects after confirmation of the underlying diagnosis. All subjects provided informed consent for tooth collection (IRB#2009-13905 at Lurie Children's Hospital). All 6 ROHHAD subjects have received a ROHHAD diagnosis. 8 of 11 patients were evaluated clinically at Lurie Children's Hospital and their diagnoses confirmed with CCHS-related *PHOX2B* mutations in CCHS subjects (*n* = 5) and with clinical phenotyping in ROHHAD subjects (*n* = 3). The excised deciduous teeth were broken to reveal the pulp, cells cultured and cell lines frozen during early passages for our DPSC Repository as previously described (21). The DPSC Repository and molecular studies on DPSC-derived neurons were approved by the UTHSC institutional review board prior to conducting research (IRB #10-00878-XP).

### 2.2. Generation of dental pulp stem cell cultures

DPSC used in this study were isolated and cultured according to our previously described protocol and cell lines stored in the DPSC Repository (21). Briefly, the dental pulp was removed from inside the tooth cavity and minced. Following removal, the pulp was digested with 3 mg/ml Dispase II and 4 mg/ml Collagenase I for 1 h. Cells were then seeded on poly-D-Lysine coated 12-well plates with DMEM/F12 1:1, 10% fetal bovine serum (FBS), 10% newborn calf serum (NCS), and 100 U/ml penicillin and 100 ug/ml streptomycin (Pen/Strep) (Fisher Scientific, Waltham, MA). Once confluent (80%) cultures were passaged using TrypLE™ Express. Only early passage cells (≤passage 4) were used for subsequent neuronal differentiation and molecular studies.

### 2.3. Neuronal differentiation

DPSC lines were seeded at 20,000 cells/cm^2^ on poly-D-lysine coated flasks with DMEM/F12 1:1, 10% fetal bovine serum (FBS), 10% newborn calf serum (NCS), with 100 U/ml penicillin and 100 ug/ml streptomycin (Pen/Strep). At 80% confluence, the neuronal differentiation protocol was followed as previously published in Kiraly et al., 2009 (37) with an extended maturation phase (4 weeks vs. 7 days) (21). Briefly, DPSC were placed in 10 μM 5-azacytidine (Acros Scientific, Geel, Belgium) in DMEM/F12 containing 2.5% FBS and 10 ng/ml bFGF (Fisher Scientific, Waltham, MA) for 48 h. Following epigenetic reprogramming, neuronal differentiation was induced by exposing the cells to 250 μM IBMX, 50 μM forskolin, 200 nM TPA, 1 mM db-cAMP (Santa Cruz, Dallas, TX), 10 ng/ml bFGF (Invitrogen, Carlsbad, CA), 10 ng/ml NGF (Invitrogen, Carlsbad, CA), 30 ng/ml NT-3 (Peprotech, Rocky Hill, NJ), and 1% insulin-transferrin-sodium selenite premix (ITS) (Fisher Scientific, Waltham, MA) in DMEM/F12 for 3 days. At the end of the neural induction period, neuronal maturation was performed by maintaining the cells in Neurobasal A media (Fisher Scientific, Waltham, MA) with 1 mM db-cAMP, 2% B27, 1% N2 supplement, 30 ng/ml NT-3, and 1X Glutamax (Fisher Scientific, Waltham, MA) for 28 days.

### 2.4. RNA sequencing of DPSC-neurons

After neuronal maturation for 28 days, total RNA was collected from the DPSC-neurons using the Zymo Directzol RNA extraction kit (Zymo, Irvine, CA). Prior to sequencing, RNA was assayed for integrity and quality using the Agilent Bioanalyzer 6000 pico chip (Agilent, Santa Clara, CA). Only RNA with an RNA Integrity Number (RIN) ≥9.0 was used for RNAseq studies. Library preparation and RNAseq was performed by Novogene (NovaSeq 6000 PE150) (Sacramento, CA) using the Illumina platform and paired end reads. 20 M paired-end reads per sample were collected.

### 2.5. RNAseq analysis

FASTQ files from Novogene were analyzed for quality and trimmed using FASTQC. All reads were trimmed to remove nucleotides with Phred scores <Q20. The trimmed FASTQ files were aligned to the human genome reference library hg19 using RNASTAR (45). Once aligned, the SAM files were collected and mined for read count information of each gene present in the reference file. Read counts were normalized using Counts per Million (CPM) method across groups for the entire experiment. Principle component analysis and Pearson's coefficient plots were performed on the normalized transcriptome profile. A lmfit and voom was used to perform differential expression analysis (45). All genes that fail to yield a *p*-value ≤0.05 and a fold change greater than 1.5 were removed. Benjamini and Hochberg false discovery rate (FDR) was performed on this trimmed gene list. All genes that failed to yield an FDR rate of ≤0.05 were removed. The PWS RNAseq dataset used here is from our previously published work (44) and accessible through the GEO database (GEO#: GSE178687). To generate Venn diagrams the final list of significantly differentially expressed genes were uploaded into an online Venn diagram tool (http://bioinformatics.psb.ugent.be/webtools/Venn/). Heatmaps were created using the ClustVis web tool (46). Additionally, the targets were loaded into the web based enrichment analysis tool, Gene Ontology Enrichment Analysis and Visualization Tool (GOrilla) to identify enriched gene ontology (GO) terms (47). The GOrilla software takes the full list of transcripts ranked by False Discovery Rate (FDR) and identifies GO terms that appear densely at the top of the ranked list. GOrilla assigns an enrichment score based on the total number of genes in the dataset (N), the total number of genes associated with a specific GO term (B), the number of genes in the top of the dataset (n), and the number of genes in the intersection (b). The enrichment score is calculated by the formula: (b/n)/(B/N).

### 2.6. Western blots

Protein was collected from 4-week mature neuronal cultures using N-PER Protein Extraction Reagent (Fisher Scientific, Waltham, MA) supplemented with a protease inhibitor cocktail (Fisher Scientific, Waltham, MA). Samples were resolved on a 4%–12% Bis-Tris gel run at a constant 125 V for 3 h, then transferred for 23 min onto a PVDF membrane using Genscript eBlot machine (L00686). Membranes were blocked at room temperature for 1.5 h in TBS-T with 5% nonfat milk and incubated at 4°C overnight in primary antibodies at 1:2,000. Primary antibodies used: α-ADORA2A (ProteinTech, 51092-1-AP) and α-PHACTR1 (ProteinTech, 23446-1-AP), and loading control α-GAPDH (Santa Cruz, sc-47724). Following overnight incubation with primary antibodies, three 25-minute washes with TBS-T were performed and membranes were incubated with secondary antibodies for 30 min at room temperature. Secondary antibodies used: HRP-conjugated α-rabbit (Cell Signaling, 7074P2) at 1:5,000 dilution and HRP-conjugated α-mouse (Cell Signaling, 7076P2) at 1:10,000 dilution. Following secondary antibody incubation, three 25-minute washes in TBS-T were performed followed by a final 25-minute wash in TBS. Pierce ECL Western (Fisher Scientific, Waltham, MA) reagents were used for GAPDH exposure. SuperSignal West Atto Ultimate Sensitivity ECL (Fisher Scientific, Waltham, MA) reagents were used to develop the ADORA2A and PHACTR1 membranes. Developed films were then digitized as non-compressed.tiff files using a V600 Epson scanner. Files were processed using ImageJ Fiji using the gel quantification plugin. The mean control sample measurements were used to normalize each lane for ADORA2A and GAPDH blots respectively. ADORA2A was then normalized against GAPDH for final protein fold change values. These values were then analyzed in Prism software (GraphPad).

## 3. Results

### 3.1. Molecular and phenotypic overlap between ROHHAD, CCHS, and PWS

Table 1 lists the defining ROHHAD features and the presence or absence of these in CCHS and PWS. The rapid-onset obesity, although a less steep trajectory in PWS and differing age at onset, is present in both ROHHAD and PWS. Additionally, PWS and ROHHAD share hypothalamic dysfunction including growth hormone insufficiency, hypothyroidism, and altered pubertal onset (precocious in ROHHAD but delayed in PWS). ROHHAD and CCHS share the hypoventilation phenotype which is the most severe symptom in both syndromes as well as neural crest tumors in a subset of individuals. All three syndromes display ANS dysregulation including gastrointestinal issues and ophthalmologic manifestations.

**Table 1:** Overlapping clinical phenotypes of ROHHAD, CCHS, and PWS. Data for ROHHAD and CCHS taken from Ceccherini, I., et al. (2022). “Developmental disorders affecting the respiratory system: CCHS and ROHHAD.” Handb Clin Neurol 189: 53–9. PWS data taken from Barclay, S. F., et al. (2018). “ROHHAD and Prader-Willi syndrome (PWS): clinical and genetic comparison.” Orphanet J Rare Dis 13(1): 124. Rapid-onset obesity with hypothalamic dysfunction, hypoventilation, and autonomic dysfunction (ROHHAD), Congenital Central Hypoventilation syndrome (CCHS).

Clinical Observation	ROHHAD	CCHS	PWS
Obesity	Yes	No	Yes
Hypoventilation	Yes	Yes	Sometimes
Hypothalamic dysfunction	Yes	Rarely	Yes
Hypothyroidism	Sometimes	Rarely	Sometimes
Growth hormone insufficiency	Yes	Rarely	Yes
Altered pubertal onset	Sometimes	No	Sometimes
Autonomic dysfunction	Yes	Yes	Yes
Bradycardia	Sometimes	Sometimes	No
Gastrointestinal dysfunction	Yes	Yes	Sometimes
Hirschsprung disease	No	Often	No
Neural crest tumors	Yes	Sometimes	No
Ophthalmologic manifestations	Yes	Yes	Yes
Altered pain perception	Yes	Yes	Yes
Thermal dysregulation	Yes	Yes	Yes
Seizures	Sometimes	Sometimes	Sometimes
Neonatal hypotonia	No	Sometimes	Yes
Neurocognitive delay	Rarely	Variable	Yes

**Figure 1:**
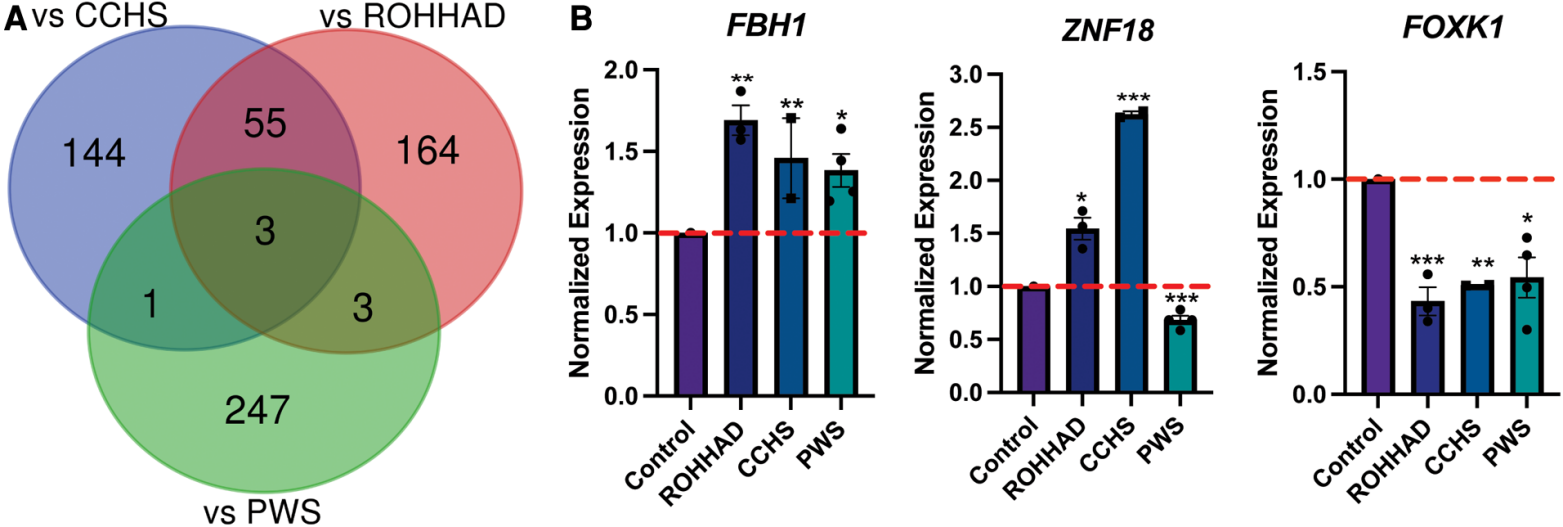
ROHHAD, CCHS, and PWS neurons share 3 differentially regulated genes vs. control neurons. (**A**) Venn diagram of the significantly differentially expressed transcripts (*p*-value ≤ 0.05, FDR ≤ 0.05, fold-change ≤0.5 or ≥1.5) vs. control for ROHHAD, CCHS, and PWS. Three transcripts were identified as differentially regulated vs. control subjects in all 3 syndromes, while 55 differentially expressed transcripts were shared between ROHHAD and CCHS vs. control. (**B**) Average RNAseq expression for each group across the three 3 genes shared by ROHHAD, CCHS and PWS vs. controls. Expression was normalized to the average of the control expression in each study. The dashed red line indicates control expression level. PWS data were gathered from our previous RNAseq experiment (44). Significance determined during RNAseq data analysis (*p*-value ≤ 0.05 and FDR ≤ 0.05). * = *p*-value ≤ 0.05, ** = *p*-value ≤ 0.01, *** = *p*-value ≤ 0.005. Rapid-onset obesity with hypothalamic dysfunction, hypoventilation, and autonomic dysregulation (ROHHAD), Congenital Central Hypoventilation Syndrome (CCHS), Prader-Willi Syndrome (PWS).

To understand the molecular similarities and differences among these three syndromes, RNA sequencing (RNAseq) analysis was performed on DPSC-derived neuronal cultures from neurotypical controls (*n* = 3) and subjects with ROHHAD (*n* = 3) or CCHS (*n* = 2). Throughout this work, we use the term transcript to describe gene-level expression differences. Table 2 lists the GUIDs and clinical characteristics for each subject used in the molecular studies (RNAseq and western blots). For the PWS dataset, we used our previously published RNAseq data from DPSC-neurons (GSE178687) (44). Differential gene expression analysis for each of the syndromes vs. the neurotypical control subjects were analyzed by the UTHSC Bioinformatics Core and significantly (*p*-value ≤0.05, FDR ≤0.05, Fold change ≥1.5 or ≤0.5) differentially expressed transcripts were identified. Using this list of significantly different transcripts vs. control neurons as well as our previously published RNAseq data from PWS neurons vs. control (*n* = 4) (44), we created a Venn diagram (Figure 1A). Venn analysis shows ROHHAD neurons have their own unique signature, but also share molecular commonalities with CCHS and PWS. In addition, our data shows that these syndromes share three significantly differentially expressed transcripts vs. control. These transcripts are *FOXK1*, *ZNF18*, and *FBH1*. The normalized RNAseq expression for these shared transcripts across syndromes is shown in Figure 1B. The RNAseq data for each experiment was normalized to the average of the neurotypical control samples in the corresponding experiments. *FOXK1* and *ZNF18* are both transcriptional regulators. *FBH1* is critical for repairing stalled or damaged replication forks during DNA replication. Broadly, these results indicate very little overlap among all three syndromes, but significant overlap between CCHS and ROHHAD gene expression signatures. It should be noted that, although each experiment was normalized to neurotypical control expression, the PWS experiment occurred separately and, as such, batch effects may be present.

**Table 2:** Subjects included in molecular experiments listed by GUID (https://nda.nih.gov/general-query.html) with corresponding clinical phenotypes. For 3 ROHHAD subjects, we have limited clinical data. *PHOX2B* expansion number listed in parentheses. Age of onset listed in years. Type of neural crest tumor: GN (ganglioneuroma) or NB (neuroblastoma).

GUID	Experiment	Diagnosis	Sex	Age of Onset	Ventilatory Support	Seizures	Cardiac Arrest	Neural Crest Tumor
GRDRMK467CLT	RNAseq	ROHHAD	F	4	Awake/Asleep	Yes	No	Yes (GN)
GRDRJR976DWY	RNAseq	ROHHAD	M	6	Asleep	Yes	No	No
GRDRPN312JYM	RNAseq/Western Blot	ROHHAD	M	1.5	Asleep	No	No	No
NIH-INVYZ061FERBJ	Western Blot	ROHHAD	F	4	Asleep	Yes	No	Yes (GN)
GRDRUD154GNH	Western Blot	ROHHAD	M	2.6	Asleep	No	No	No
GRDRGZ805WLY	Western Blot	ROHHAD	M	3.5	Asleep	No	No	No
GRDRHC489JUK	RNAseq	CCHS (20/33)	M	Neonate	Asleep	Yes	No	No
GRDRPU870HZG	RNAseq/Western Blot	CCHS (20/25)	F	Neonate	Asleep	Yes	No	No
GRDRHC22YV2	Western Blot	CCHS (20/26)	F	Neonate	Awake/Asleep	Yes	Yes	No
GRDRWX199GXE	Western Blot	CCHS (20/27)	F	Neonate	Awake/Asleep	Yes	No	No
NIH-INVLG921GKWXE	Western Blot	CCHS (20/33)	M	Neonate	Awake/Asleep	No	No	Yes (NB)

### 3.2. Gene ontology enrichment in receptor, acetylglucosaminyltransferase and immune-mediated processes in ROHHAD neurons

**Figure 2:**
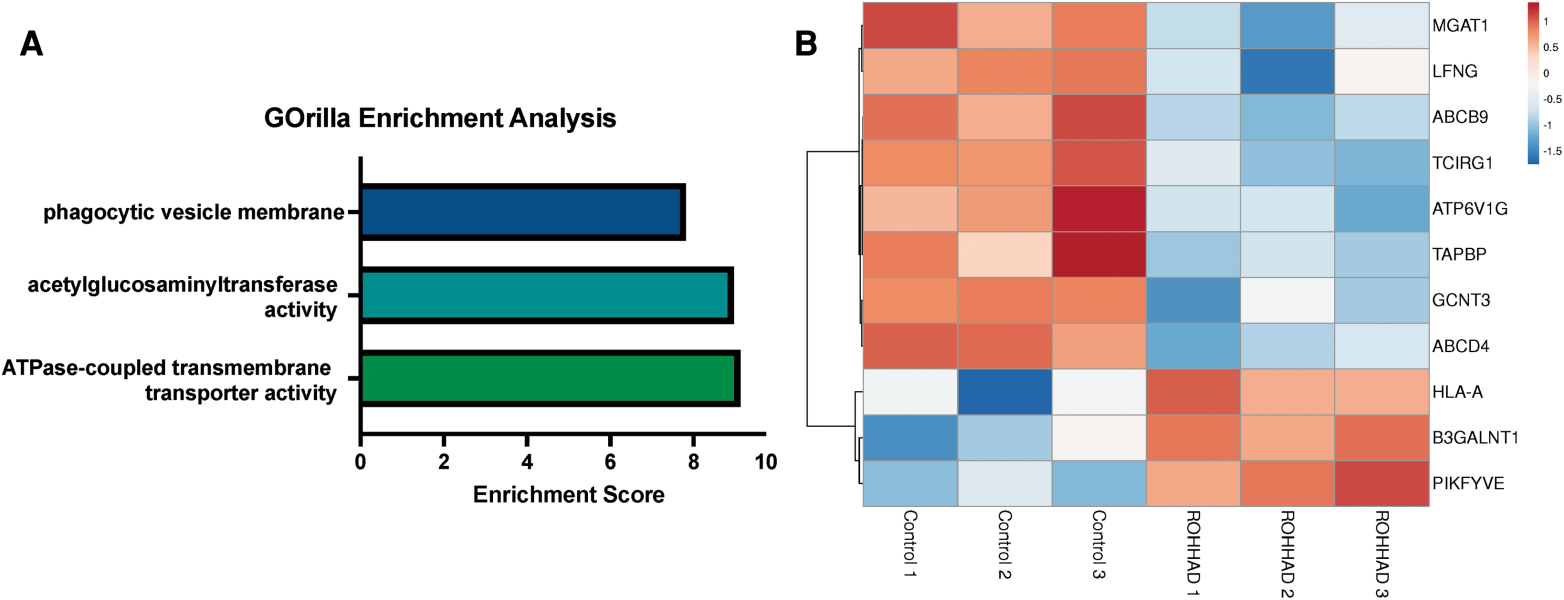
Enrichment analysis of ranked gene expression differences between ROHHAD and control neurons shows significant enrichment in phagocytic vesicle membrane, acetylglucosaminyltransferase activity and transmembrane transporter activity. (**A**) GOrilla enrichment analysis was used to determine the gene ontology (GO) enrichments in our Control vs. ROHHAD dataset (47). GOrilla analysis identified 3 GO categories (enrichment score ≥ 5) that are significantly enriched (*p*-value ≤ 0.05) at the top of our ranked gene list. (**B**) A heatmap was created using the RNAseq expression counts for each ROHHAD and control individual for genes identified in each of the enrichment categories using ClustVis (46).

RNAseq analysis identified 225 genes to be significantly differentially expressed in ROHHAD vs. control neurons. Using GOrilla enrichment analysis, we found significant gene ontology (GO) enrichments in these data. GOrilla enrichments are presented in Figure 2A. The top enrichments (enrichment score ≥5) were ATPase transmembrane transporter activity (*ATP6V1G2*, *ABCB9*, *TAPBP*, *ABCD4* and *TCIRG1*), acetylglucosaminyltransferase activity (*MGAT1*, *GCNT3*, *LFNG* and *B3GALNT1*) and phagocytic vesicle membrane (*HLA-A*, *TAPBP*, *PIKFYVE* and *TCIRG1*). A heatmap of the genes found in these enrichments across each subject sequenced is shown in Figure 2B. Most of these genes have significantly reduced expression vs. control, while three show increased expression. Gene functions are listed in Supplementary Table S1 (48). Several of the genes identified in the enrichment categories relate to immune system processes and neuroinflammatory syndromes. These enrichment studies on ROHHAD specific neuronal gene expression indicate that at least three molecular pathways may contribute to disease etiology: ATPase transmembrane transporters, acetylglucosaminyltransferases, and phagocytic vesical membrane proteins.

### 3.3. Molecular overlap between PWS and ROHHAD May not be related to rapid-onset obesity

The RNAseq data collected in this experiment compared with our previously published RNAseq data (44) revealed six genes that are significantly different in both PWS and ROHHAD vs. control. The Venn diagram in Figure 1A shows this overlap. Three of the six genes found in this intersection are unique to PWS and ROHHAD and are not significantly different in the CCHS vs. control dataset. These genes are *ID1*, *CNN3*, and *OAZ3*. To compare expression across two datasets, the RNAseq data for ROHHAD and PWS subjects was normalized to the average control expression of the transcript in each dataset. This normalized expression was used to create bar graphs for each gene (Supplementary Figure S1). For *ID1*, PWS and ROHHAD neurons show differing expression trends vs. control. In PWS, *ID1* expression is significantly higher, while in ROHHAD expression is significantly lower. One of the primary reasons for comparing PWS to ROHHAD in this experiment was to see if any of the overlapping genes were related to the rapid-onset obesity phenotype seen in each syndrome. The three overlapping genes in our dataset do not currently have any links to an obesity pathogenesis. *ID1* is a transcriptional regulator. *OAZ3* plays a role in cell proliferation and maintenance. *CNN3* is involved in cytoskeletal function and is actin-binding. These results suggest that the obesity in ROHHAD may not share a molecular pathway with PWS and may be distinct in origin. Further validation of this hypothesis in other relevant cell types will be necessary to confirm the independent obesogenic pathology in PWS and ROHHAD.

### 3.4. Gene expression analysis reveals shared molecular signature between ROHHAD and CCHS neurons

**Figure 3:**
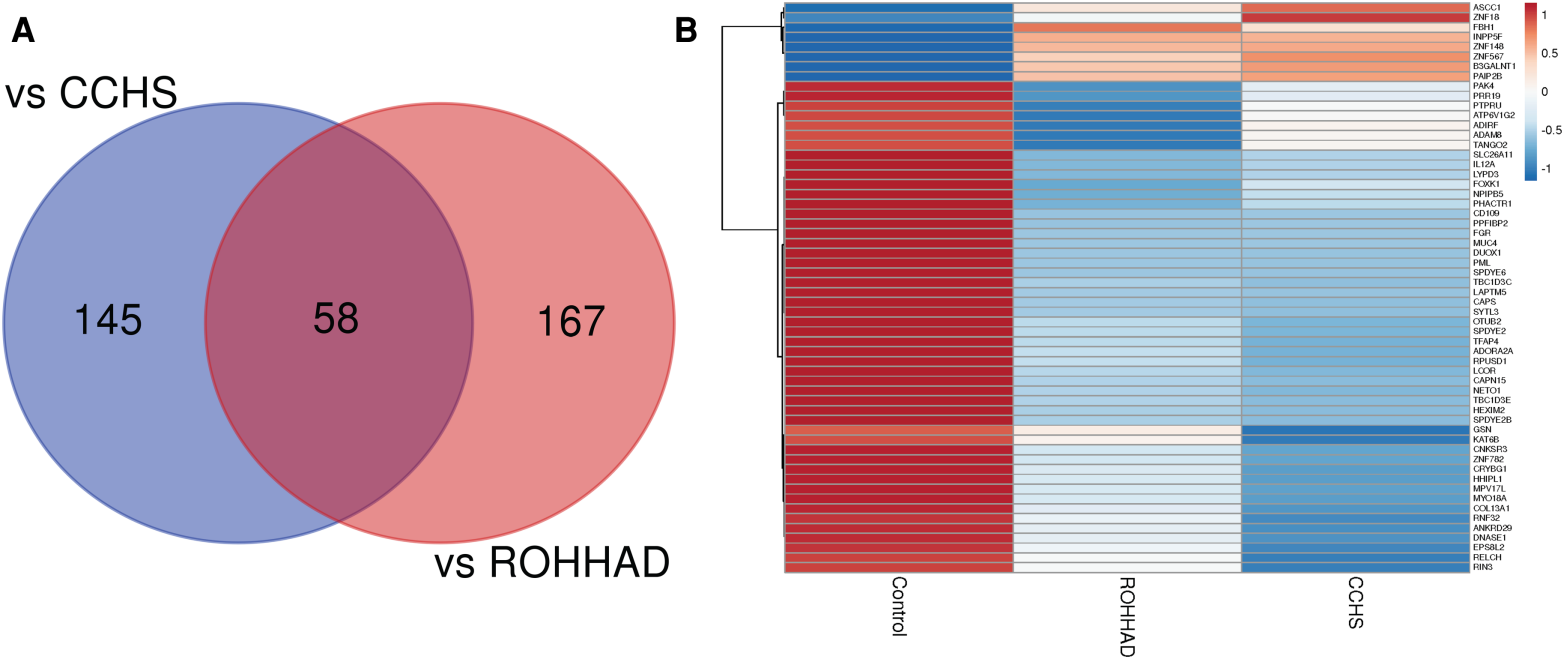
ROHHAD and CCHS share 58 differentially expressed genes vs. neurotypical control subjects. (**A**) Venn diagram created using the list of differentially expressed transcripts (*p*-value ≤ 0.05, FDR ≤ 0.05, fold-change ≤0.5 or ≥1.5) vs. control subjects. (**B**) Heatmap of the 58 overlapping transcripts differentially expressed in ROHHAD and CCHS neurons vs. control neurons. Heatmap was created using ClustVis (46).

The Venn diagram in Figure 3A shows that there are 58 genes significantly differentially expressed in both ROHHAD and CCHS vs. control. Using the mean RNAseq counts per group, a heatmap was created using ClustVis (46). The heatmap (Figure 3B) indicates that most of these genes are significantly decreased vs. control in both ROHHAD and CCHS. Eight of these genes show higher expression in ROHHAD and CCHS vs. control. For all genes identified in this overlap, the expression trend vs. control is the same for both ROHHAD and CCHS. A list of these genes and their function is listed in Supplementary Table S2 (48). Several of the genes identified encode for proteins that have functions related to neuronal development, including *ADAM8*, *KAT6B* and *ADIRF*. Additionally, *ASCC1*, *COL13A1*, *SYTL3*, *TANGO2*, *PHACTR1*, and *ADORA2A* encode proteins that have functions related to ROHHAD and CCHS phenotypes. These expression studies have revealed molecular overlap between ROHHAD and CCHS neurons vs. neurotypical controls. This implies that similar or the same pathways may be disturbed in both syndromes leading to shared phenotypic characteristics.

### 3.5. ADORA2A protein does not consistently change in ROHHAD but shows reduction in CCHS with increasing *PHOX2B* PARM number

**Figure 4:**
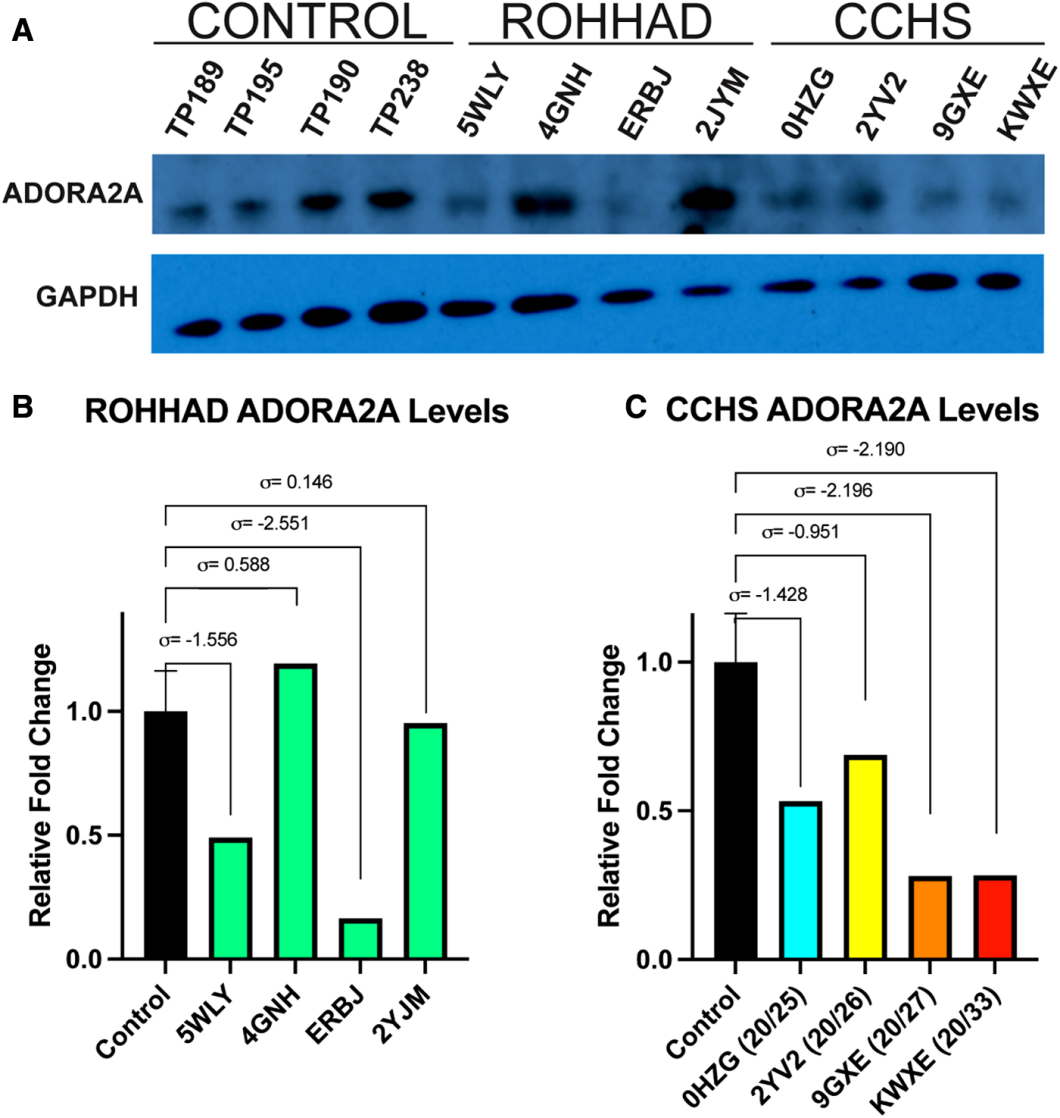
Western blot analysis of ADORA2A reveals differential expression in ROHHAD and CCHS neurons. (**A**) ECL western blot of ADORA2A in Control, ROHHAD, and CCHS groups. An average of all four controls was used for normalization and comparison to each of the four individual ROHHAD and the four individual CCHS cell lines. The graphs were scaled differently for ROHHAD vs. CCHS comparisons, but the same control samples and averages were used in B and C. (**B**) Quantification of ADORA2A protein in ROHHAD individuals. Standard deviation values (*σ*) are derived from the average of the control samples (*n* = 4). 5WLY and ERBJ show a reduction in ADORA2 protein, 4GNH shows a nominal increase in protein expression and 2JYM remains near control expression. (**C**) ADORA2A quantification in CCHS individuals. 0HZG (20/25) and 2YV2 (20/26) show a reduction in ADORA2A expression. 9GXE (20/27) and KWXE (20/33) show a dramatic reduction in signal >2 standard deviations from the control average value.

One gene that changed at the transcript level in neurons and could play a role in both ROHHAD and CCHS is the *ADORA2A* gene, which encodes an adenosine receptor. *ADORA2A* is a strong candidate for validation due to its link to Parkinson disease and functional overlap with CCHS phenotypes. We used western blot analysis of ADORA2A protein in ROHHAD and CCHS DPSC-derived neurons vs. neurotypical controls to verify that changes in gene expression are reflected at the protein level (Figure 4A). There was no consistency amongst ROHHAD individuals for ADORA2A protein regulation (Figure 4B). Two individuals, 5WLY and ERBJ, showed reduction (∼55% and ∼85%) in ADORA2A levels compared to the average control groups. One individual, 4GNH, showed an increase (∼132%) in ADORA2A. The remaining ROHHAD individual, 2JYM, displayed ADORA2A protein expression close to control levels.

CCHS individuals exhibited a decrease in ADORA2A protein expression that correlated to an increase in PARM number. The 20/25 PARM individual (0HZG) showed a reduction (∼51%) in ADORA2A protein. Whereas the 20/26 (2YV2) individual showed a reduction of 23%. The PARM 20/27 (9GXE) and 20/33 (KWXE) individuals showed a reduction (∼62%) of ADORA2A protein, both raw expression values for these two samples were >2 standard deviations from the average control expression value (Figure 4C). Supplementary Figure S2 shows an additional ADORA2A western blot using a 10% Bis-Tris gel and nitrocellulose membrane performed prior to the optimized blot shown in Figure 4. Sample TP312 was replaced with sample TP195 in Figure 4. We also examined another protein of interest, PHACTR1, however, it showed no consistency amongst controls, ROHHAD, and CCHS DPSC-derived neuron lines (Supplementary Figure S3). These studies may indicate a possible trend between decreasing ADORA2A expression and more severe CCHS genotypes (i.e., PARMS ≥20/27). Further validation in a larger cohort of CCHS subjects with varying PARM mutations will be necessary to confirm PHOX2B-mediated regulation of ADORA2A.

## 4. Discussion

Uncovering the molecular commonalities and differences among these three syndromes is a crucial next step for identifying therapeutics and biomarkers specific to ROHHAD. Here, we used our unique model system, DPSC-derived neurons, to investigate molecular perturbations in these syndromes vs. neurotypical children and identify unique and overlapping transcriptional signatures. Previously, we used DPSC neuronal cultures to investigate neurogenetic syndromes including Prader-Willi, Angelman, and Duplication 15q syndrome (42–44). In this study, using RNAseq from individuals with ROHHAD, CCHS, and neurotypical children, we identified differentially expressed transcripts with shared expression between ROHHAD and CCHS. We then used previously published RNAseq data from PWS DPSC-derived neuronal cultures to compare these syndromes to PWS subjects (44). We identified gene ontology enrichments in ROHHAD neurons, three transcripts that are differentially expressed in all three syndromes vs. neurotypical control neurons and 58 transcripts that are differentially expressed in both ROHHAD and CCHS neurons.

For ROHHAD, CCHS, and PWS the three transcripts that are significantly different between all three syndromes vs. control neurons were *FOXK1*, *FBH1*, and *ZNF18*. All three genes play roles in maintaining DNA integrity or transcriptional regulation.

### 4.1. FOXK1

*FOXK1* is crucial in regulating glucose metabolism and aerobic glycolysis (49, 50). Compared to control neurons, ROHHAD, CCHS, and PWS neurons all had reduced expression of *FOXK1*. The reduction in expression in neurons from these three syndromes may pertain to a delay in neurogenesis, a feature shared among many developmental disorders. As stem cells develop into neurons, they switch their primary metabolic processes from glycolysis to mitochondrial respiration (51). *FOXK1* plays a key role in this process (50). Additionally, *FOXK1* has been associated with autism and delayed development in several studies (52, 53).

### 4.2. FBH1

*FBH1* plays a role in responding to stalled or damaged replication forks. In response to DNA damage, *FBH1* causes DNA double-stranded breaks and induces cell death (54–56). ROHHAD, CCHS, and PWS have increased *FBH1* expression compared to control neurons. The increased expression of *FBH1* may be caused by or causative of increased DNA damage in disease neurons. Studies have shown that *FBH1* is required for eliminating cells with excessive replicative stress (54). Additionally, accumulation of damaged DNA has been directly linked to neuronal death in other syndromes, such as Alzheimer's disease (57, 58).

### 4.3. ZNF18

*ZNF18* is involved in regulating transcription through RNA polymerase II. Although the function of this protein has not been well characterized, mutations in this gene have been associated with autism (59). This association may explain the differing directionality in expression for ROHHAD and CCHS vs. PWS, where the incidence of autism spectrum disorder is higher (60, 61). Expression of *ZNF18* in PWS neurons is significantly decreased vs. control. In contrast, *ZNF18* expression is significantly increased in ROHHAD and CCHS neurons.

Enrichment analysis using the ranked list of differentially expressed transcripts between ROHHAD vs. neurotypical control neurons identified significant enrichments in the phagocytic vesicle membrane compartment (GO:0045335), transmembrane transporter activity (GO:0042626), and acetylglucosaminyltransferase activity (GO:0008375). The four transcripts identified in the phagocytic vesicle membrane enrichment were *HLA-A*, *TAPBP*, *PIKFYVE* and *TCIRG1.* A phagocytic vesicle is an intracellular vesicle that arises due to phagocytosis, a key cellular process for eliminating cellular waste and maintaining cellular homeostasis (62). In neurons, dysfunctional phagocytosis, either excessive or not enough, is detrimental to neuronal development and health (63). Excessive microglial phagocytosis of live neurons and synapses has been implicated in various neurodegenerative disorders including Alzheimer's and Parkinson's disease (64, 65).

In ROHHAD neurons, there was an increased expression in the *HLA-A* and *PIKFYVE* transcripts and decreased expression of the *TAPBP* and *TCIRG1* genes. *HLA-A* encodes the antigen-presenting major histocompatibility complex class I, A. This molecule plays a critical role in the immune system by presenting peptides for recognition to cytotoxic T cells (48). Inhibition of PIKFYVE, an endosomal kinase, was shown to reduce excitotoxicity and restore lysosomal maturation, in a cell model of Amyotrophic Lateral Sclerosis (66). Most transcripts identified in the acetylglucosaminyltransferase activity enrichment (*MGAT1*, *GCNT3*, *LFNG* and *B3GALNT1)* were decreased in the ROHHAD neurons. Acetylglucosaminyltransferase activity is crucial for glycosylation, an important and complex post translational modification crucial for regulation of neuronal intercellular signaling. In *Drosophila melanogaster*, *Mgat1* null mutants were found to have disrupted synaptogenesis and signaling (67). Interestingly, *MGAT1* variants are associated with susceptibility to obesity (68). Decreased glycosylation activity is associated with neuronal death and neurodegenerative disease (69).

All transcripts identified in the transmembrane transporter activity category were decreased in the ROHHAD neurons. Several of the protein products of these transcripts localize to lysosomes. Neuronal lysosomes are crucial for degrading cellular debris and maintaining neuronal integrity. All the enrichment data taken together may allude to a dysfunction in clearing cellular debris and leading to decreased neuronal health in affected neurons or an immune mediated process related to ROHHAD and the propensity for ROHHAD neural crest cells to migrate and develop neural crest tumors in the body as DPSC are of neural crest origin.

We identified three transcripts that were uniquely significantly different between ROHHAD and PWS vs. control (*ID1*, *CNN3*, and *OAZ3*). Although one of the primary commonalities between these syndromes is childhood obesity, these three genes do not appear to be related to obesity and two of these genes appear to have opposing expression patterns vs. control neurons. Our molecular findings indicate that while both ROHHAD and PWS subjects present with childhood obesity, it is likely that the pathways responsible for obesity in these disorders arise from distinct molecular and perhaps even behavioral defects. Lending support to this theory, PWS subjects are profoundly hyperphagic which is not routinely an underlying cause of obesity in ROHHAD patients (3).

Of note, we identified 58 significantly differentially expressed transcripts vs. neurotypical controls in common between CCHS and ROHHAD, all following similar expression patterns [i.e., both syndromes show either increased or decreased expression for the same gene vs. control (see Figure 3B)]. Within the 58 genes, we did not find any significant gene ontology enrichments, however, we did identify transcripts that correlate with neuronal processes and the phenotypes observed in both ROHHAD and CCHS, including *ASCC1*, *COL13A1*, *TANGO2*, and *PHACTR1*.

### 4.4. ASCC1

*ASCC1*, a gene encoding for a transcriptional regulator, was found to be significantly increased in ROHHAD and CCHS neurons. This transcript has been associated with several congenital neuromuscular diseases including spinal muscular atrophy (70–72).

### 4.5. COL13A1

*COL13A1*, which encodes an extracellular synaptic protein that is required for neuromuscular junction synapse function, was significantly decreased in ROHHAD and CCHS neurons. Mutations in this gene are associated with a spectrum of myasthenic diseases that result in breathing difficulties and apneas (73–75).

### 4.6. TANGO2

*TANGO2* mutations and deficiency are associated with a variety of symptoms including hypothyroidism, metabolic and cardiac dysfunction, and rhabdomyolysis (76–79). This gene was found to be significantly decreased in our dataset for both ROHHAD and CCHS vs. control neurons.

### 4.7. PHACTR1

*PHACTR1* encodes a phosphatase and actin regulatory protein that plays a role in neuronal migration and dendritic arborization. Although we did see a reduction in PHACTR1 protein by western blot in several of the ROHHAD and CCHS subjects (Supplementary Figure S3) in accordance with our RNAseq data, this result was variable from individual to individual.

An adenosine receptor gene, *ADORA2A*, was also found to be decreased in the ROHHAD and CCHS neurons vs. neurotypical controls. *ADORA2A* encodes a member of a G-protein coupled receptor family that functions by increasing cAMP levels using adenosine as its primary agonist and is associated with Parkinson's disease (80, 81). Physiologically, ADORA2A aides in regulation of cardiac rhythm and circulation, cerebral and renal blood flow, immune function, pain regulation and sleep. In *Adora2a*^−/−^ mice, Adora2a specifically modulates the antiadrenergic effects of the Adora1 receptor (82). Treatment with isoproterenol, a *β*-adrenergic molecule agonist, in WT and *Adora2a*^−/−^ mice both showed antiadrenergic responses, with *Adora2a*^−/−^ mice exhibiting a ∼40% increase in antiadrenergic response. These findings supplement prior work showing decreased heart rate (HR) in *Adora2a*^−/−^ mice and an increase in HR for *Adora1*^−/−^ mice (83). Previous clinical studies on ROHHAD patients show that some cases may present with bradycardia, a low resting heart rate (84). The observed reduction of ADORA2A protein levels in two ROHHAD individuals may be able to explain this clinical feature. ADORA2A is thought to provide a protective effect against the development of pulmonary hypertension (85), another clinical phenotype that presents in some ROHHAD individuals consequent to recurrent low oxygen due to inadequately managed hypoventilation (5, 86). Our findings that CCHS individuals have decreasing ADORA2A protein expression with increasing *PHOX2B* PARM number suggests that ADORA2A regulation may be dependent on proper PHOX2B activity. In fact, two *PHOX2B* transcription factor binding sites can be found in or near the promoter of the *ADORA2A* gene (Supplementary Figure S4). The variance seen in the ROHHAD subjects for ADORA2A protein expression, as well as PHACTR1, is likely due to the spectrum of ROHHAD symptomology that unfolds across time and with varying severity. Further studies will be needed to establish that PHOX2B can directly regulate *ADORA2A* gene expression levels, which could be an important finding in both ROHHAD and CCHS.

The results presented here represent the first study using primary ROHHAD and CCHS neurons and showing molecular correlation between CCHS and ROHHAD pathogenesis. Although groundbreaking, there are some limitations to the interpretation of the data. As this is a rare syndrome, we were limited in the amount of specimen used for both RNAseq and western blot analysis. Further validation of these results in larger cohorts of ROHHAD and CCHS subjects will be necessary to confirm the results presented here. The PWS RNAseq data used for comparison here came from a separate experiment and, as such, batch effects may make comparing to the current study problematic. Additionally, these are developmentally young cortical-like neurons. However, the early neurodevelopmental timeframe that this model represents provides evidence of perturbed pathways seen even in immature neurons and provides insight into disease pathogenesis that is present from early neurodevelopment and may have significant downstream effects on later developmental phenotypes.

## 5. Conclusions

ROHHAD is a complex ANS disorder with an unknown genetic etiology. Although molecular, genetic, and clinical studies have been performed on ROHHAD and CCHS patients before, this is the first gene expression study on neurons derived from these individuals. In addition, no molecular or cellular pathways had previously been identified that reveal the underlying disease etiology. Here we uncovered an enrichment in differentially expressed transcripts related to phagocytic vesicle and receptor-mediated processes in ROHHAD neurons and an overlapping molecular signature between both ROHHAD and CCHS, a related ANS hypoventilation disorder. This overlap includes transcripts that control neurodevelopmental processes and molecular pathways that may be perturbed leading to ROHHAD pathology. We then validated the expression of a down-regulated transcript, *ADORA2A,* at the protein level and found a reduction in ADORA2A protein for CCHS neurons and several ROHHAD subjects, although expression appears to vary more in the ROHHAD cohort. This variability in ROHHAD subjects likely reflects the broad spectrum of ROHHAD symptoms, the unfolding of the phenotype over time, and the unknown etiology of the disease. The data presented here describes important preliminary findings that warrant further validation in other CCHS affected individuals. Continuing molecular studies and correlation with clinical phenotypes will be essential to uncovering the molecular pathways behind the various ROHHAD symptoms and identifying biomarkers that can be used to diagnose and treat ROHHAD early in the disease process.

## Data Availability

The data presented in the study are deposited in the NIH Gene Expression Omnibus (GEO), accession number GSE216125.
