# Supplementary material for: Analysis and comparisons of gene expression changes in patient- derived neurons from ROHHAD, CCHS, and PWS
Source: Front Pediatr. 2023 May 10;11:1090084. doi: 10.3389/fped.2023.1090084 (PMC10206321; doi:10.3389/fped.2023.1090084)
Supplement: Supplementary file 1 [file Table1.docx]

Supplementary Material

# Supplementary Tables

| **Gene** | **Gene Summary** | **Enrichment Category** |
| --- | --- | --- |
| *ABCB9* | Encodes for ATP-dependent low-affinity peptide transporter which translocates a broad spectrum of peptides from the cytosol to the lysosomal lumen for degradation. | ATPase-coupled transmembrane transporter activity |
| *ABCD4* | Encodes for a lysosomal membrane protein that transports cobalamin (Vitamin B12) from the lysosomal lumen to the cytosol in an ATP-dependent manner. | ATPase-coupled transmembrane transporter activity |
| *ATP6V1G2* | Encodes for a a component of vacuolar ATPase (V-ATPase), a multisubunit enzyme that mediates acidification of intracellular compartments of eukaryotic cells. | ATPase-coupled transmembrane transporter activity |
| *B3GALNT1* | Encodes for a member of the beta-1,3-galactosyltransferase family. Plays a role in the preimplantation stage of embryonic development. | Acetylglucosaminyltransferase activity |
| *GCNT3* | Encodes for a Glycosyltransferase that can synthesize all known mucin beta 6 N-acetylglucosaminides. | Acetylglucosaminyltransferase activity |
| *HLA-A* | Encodes for Antigen-presenting major histocompatibility complex class I (MHCI) molecule. Class I molecules play a central role in the immune system by presenting peptides derived from the endoplasmic reticulum lumen so that they can be recognized by cytotoxic T cells. | Phagocytic vesicle membrane |
| *LFNG* | Encodes for a glycosyltransferase that initiates the elongation of O-linked fucose residues attached to EGF-like repeats in the extracellular domain of Notch molecules. | Acetylglucosaminyltransferase activity |
| *MGAT1* | Encodes for a glycosyltransferase that initiates complex N-linked carbohydrate formation. | Acetylglucosaminyltransferase activity |
| *PIKFYVE* | Encodes for Dual specificity kinase implicated in myriad essential cellular processes such as maintenance of endomembrane homeostasis, and endocytic-vacuolar pathway, lysosomal trafficking, nuclear transport, stress- or hormone-induced signaling and cell cycle progression. | Phagocytic vesicle membrane |
| *TAPBP* | Encodes a transmembrane glycoprotein which mediates interaction between newly assembled major histocompatibility complex (MHC) class I molecules and the transporter associated with antigen processing (TAP), which is required for the transport of antigenic peptides across the endoplasmic reticulum membrane. | Phagocytic vesicle membrane |
| *TCIRG1* | Encodes a subunit of a large protein complex known as a vacuolar H+-ATPase (V-ATPase). The protein complex acts as a pump to move protons across the membrane. This movement of protons helps regulate the pH of cells and their surrounding environment. | Phagocytic vesicle membrane and ATPase-coupled transmembrane transporter activity |

**Supplementary Table 1.** Genes identified during enrichment analysis and their function according to GeneCards.org. Safran, M., et al. (2021). The GeneCards Suite. *Practical Guide to Life Science Databases*. I. Abugessaisa and T. Kasukawa. Singapore, Springer Nature Singapore**:** 27-56.

| **Gene Name** | **Gene Summary** |
| --- | --- |
| *ADAM8* | Encodes for an ADAM (a disintegrin and metalloprotease domain) family member, involved in cell adhesion during neurodegeneration and a target for allergic respiratory diseases. |
| *ADIRF* | Adipose Regulatory Factor protein encoding gene. Involved in PPARA gene expression and the development of the nervous system. |
| *ADORA2A* | Encodes for a guanine nucleotide-binding protein-coupled receptor (GPCR) family member. Utilizes adenosine as its endogenous agonist to increase intracellular cAMP levels. Maintains proper cardiac rhythm and circulation, cerebral and renal blood flow, immune function, pain regulation, and sleep. |
| *ANKRD29* | Gene encoding for the Ankyrin Repeat Domain-Containing Protein 29 (ANKRD29) and thought to be an integral membrane component. |
| *ASCC1* | Encodes for Activating Signal Cointegrator 1 Complex Subunit 1. ASC-1 plays a vital role in gene transactivation by multiple transcription factors. Mutations are associated with Barrett esophagus and esophageal adenocarcinoma. |
| *ATP6V1G2* | Encodes for ATPase H+ Transporting V1 Subunit G2. Multisubunit enzyme mediating acidification of intracellular compartments of eukaryotic cells. This process is needed for protein sorting, zymogen activation, receptor-mediated endocytosis, and synaptic vesicle proton gradient generation. |
| *B3GALNT1* | Encodes for a member of the beta-1,3-galactosyltransferase family. Plays a role in the preimplantation stage of embryonic development. |
| *CAPN15* | Encodes for Calpain 15, a protein that acts as a transcription factor, RNA-binding protein, and protein-protein interactions during development of the visual system. |
| *CAPS* | Encodes for the protein Calcyphosine, a calcium-binding protein involved in regulation of ion transport. |
| *CD109* | Encodes for the glycosyl phosphatidylinositol (GPI)-linked glycoprotein, CD109, acting as a negative regulator of TGF-β signaling. |
| *CNKSR3* | Encodes for Connector Enhancer of Kinase Suppressor of Ras 3. Involved in the negative regulation of the ERK1 and ERK2 cascade, peptidyl-serine phosphorylation, and the positive regulation of Na+ transport. |
| *COL13A1* | Encodes for the nonfibrillar collagen, Collagen Type XIII Alpha 1 Chain, and is found in all connective tissue-producing cells. It possesses a transmembrane domain and is documented to localize to the plasma membrane. |
| *CRYBG1* | Encodes for the Crystallin Beta-Gamma Domain Containing 1 protein and is associated with Melanoma. |
| *DNASE1* | Encodes for Deoxyribonuclease 1. Mutations are associated with Systemic Lupus Erthematosus. |
| *DUOX1* | Encodes for Dual Oxidase 1, a glycoprotein family member of the NADPH oxidase family. |
| *EPS8L2* | Encodes for EPS8 family member, EPS8 Like 2. Mutations are associated with deafness and is involved in the olfactory signaling pathway and sound sensory processing. |
| *FBH1* | Encodes for the F-Box DNA Helicase 1 protein. |
| *FGR* | Encodes for the FGR Proto-Oncogene, an Src Family Tyrosine Kinase. Functions as a negative regulator of cell migration and adhesion triggered by the beta-2 integrin signal transduction pathway. |
| *FOXK1* | Encodes for the Forkhead Box K1 protein that has DNA-binding transcription repressor activity, and involved in processes such as glucose metabolism, aerobic glycolysis, muscle cell differentiation and autophagy. |
| *GSN* | Encodes for Gelsolin, an actin-depolymerizing factor that binds to actin monomers and filaments to prevent monomer exchange. Associated with Amyloidosis and Amyloidosis, Finnish Type. |
| *HEXIM2* | Encodes for Hexamethylene Bis-Acetamide-Inducible Protein 2. Negatively regulates the kinase activity of the cyclin-dependent kinase P-TEFb. |
| *HHIPL1* | Encodes for the HHIP Like 1 protein that belongs to the glucose/sorbosone dehydrogenase family. Also contains a folate and reduced folic acid derivative binding domain. |
| *IL12A* | Encodes for Interleukin 12A, a cytokine subunit. |
| *INPP5F* | Encodes for the Inositol Polyphosphate-5-Phosphatase F. Activity is specific for phosphatidylinositol 4,5-bisphosphate and phosphatidylinositol 3,4,5-trisphosphate. |
| *KAT6B* | Encodes for the Lysine Acetyltransferase 6 B, a component of the MOZ/MORF complex. Required for RUNX2-dependent transcriptional activation and potentially involved in brain development. Mutations are associated with acute myeloid leukemias. |
| *LAPTM5* | Encodes for the Lysosomal-Associated Multitransmembrane Protein 5. May play a role in hematopoiesis. |
| *LCOR* | Encodes for the Ligand Dependent Nuclear Receptor Corepressor protein. Expressed in fetal and adult tissues. Associated with Breast Medullary Carcinoma and Uterus Carcinoma. |
| *LYPD3* | Encodes for LY6/PLAUR Domain Containing 3 protein that acts upstream or within cell-matrix adhesion. |
| *MPV17L* | Encodes for the Mitochondrial Inner Membrane Protein 17 Like protein, a negative regulator of the hydrogen peroxide biosynthetic process and mitochondrial outer membrane permeabilization involved in apoptotic signaling pathway. |
| *MUC4* | Encodes for Mucin 4, one of the major constituents of mucus, covering epithelial surfaces. |
| *MYO18A* | Encodes for the Myosin XVIIIA protein. Binds to GOLPH3, linking the Golgi to the cytoskeleton and influencing Golgi membrane trafficking, may also be required for cell migration. |
| *NETO1* | Encodes for the Neuropilin And Tolloid Like 1 protein. Thought to play a role in Hippocampal spatial learning and memory through regulation of synaptic N-methyl-D-aspartic acid receptor complexes. |
| *NPIPB5* | Encodes for the Nuclear Pore Complex Interacting Protein Family Member B5 protein. |
| *OTUB2* | Encodes for the OUT Deubiquitinase, Ubiquitin Aldehyde Binding 2 protein. Involved in protein metabolism. |
| *PAIP2B* | Encodes for Poly(A) Binding Protein Interacting Protein 2B. Enhances translation by circularizing mRNA through EIF4G1 translation initiation factor interaction. |
| *PAK4* | Encodes for P21 (RAC1) Activated Kinase 4 and acts as an effector to link Rho GTPases to the cytoskeleton during reorganization and nuclear signaling. |
| *PHACTR1* | Encodes for Phosphatase And Actin Regulator 1 protein. Functions by binding actin and regulating the actin cytoskeleton reorganization. |
| *PML* | Encodes for the PML Nuclear Body Scaffold protein, a member of the tripartite motif (TRIM) family. Functions as a transcription factor and tumor suppressor. |
| *PPFIBP2* | Encodes for PPFIA Binding Protein 2, member of the LAR protein-tyrosine phosphatase-interacting protein (Liprin) family. Plays part in axonal guidance and neuronal synapse development by LAR protein-tyrosine phosphatase plasma membrane recruitment. |
| *PRR19* | Encodes for Proline Rich 19 protein that promotes meiotic crossing over formation through CNTD1 interaction. |
| *PTPRU* | Encodes for Protein Tyrosine Phosphatase Receptor Type U, a protein tyrosine phosphatase (PTP) family member. PTPs are known to regulate cell growth, differentiation, mitotic cycle, and oncogenic transformation through signaling. |
| *RELCH* | Encodes for RAB11 Binding And LisH Domain, Coiled-Coil And Heat Repeat Containing protein. Involved in intracellular cholesterol transport. |
| *RIN3* | Encodes for Ras And Rab Interactor 3 protein and acts as a binding partner to RAB5 small GTPases. |
| *RNF32* | Encodes for Ring Finger Protein 32. RING finger motifs are observed in functionally different proteins involved in protein-DNA or protein-protein interactions. Expressed during spermatogenesis. |
| *RPUSD1* | Encodes for the RNA Pseudouridine Synthase Domain Containing 1 protein. Thought to be involved in enzyme-directed rRNA pseudouridine synthesis and enable pseudouridine synthase activity. |
| *SLC26A11* | Encodes for the Solute Carrier Family 26 Member 11 protein. Family members are crucial for cellular homeostasis and intracellular electrolyte balance. |
| *SPDYE2* | Encodes for Speedy/RINGO Cell Cycle Regulator Family Member E2. Thought to enable protein kinase binding activity. |
| *SPDYE2B* | Encodes for Speedy/RINGO Cell Cycle Regulator Family Member E2B. Thought to enable protein kinase binding activity. |
| *SPDYE6* | Encodes for Speedy/RINGO Cell Cycle Regulator Family Member E6. Plays a role in cell cycle progression through binding and activation of cylin-dependent kinases. |
| *SYTL3* | Encodes for the Synaptotagmin Like 3 protein. Functions by binding to phospholipids in the presence of Ca^2+^ ions. |
| *TANGO2* | Encodes for the Transport And Golgi Organization 2 Homolog protein. Thought to play a role in secretory protein loading in the endoplasmic reticulum. |
| *TBC1D3C* | Encodes for TBC1 Domain Family Member 3C. Thought to be involved in GTPase signaling and vesicle trafficking. |
| *TBC1D3E* | Encodes for TBC1 Domain Family Member 3E. Thought to be involved in the activation of GTPase activity and intracellular protein transport. |
| *TFAP4* | Encodes for the Transcription Factor AP-4 protein. Activated both viral and cellular genes by binding to symmetrical CAGCTG DNA sequences. |
| *ZNF18* | Encodes for Zinc Finger Protein 18. Has RNA polymerase II-specific and RNA polymerase II-cis regulatory region sequence-specific DNA binding activity. Thought to regulate transcription by RNA polymerase II. |
| *ZNF148* | Encodes for Zinc Finger Protein 148. Activates transcription of T-cell receptor and intestinal alkaline phosphatase genes. Mutations have been associated with global developmental delay, hypoplastic corpus callosum, and dysmorphic facies. |
| *ZNF567* | Encodes for Zinc Finger Protein 567. Thought to enable DNA-binding transcription factor activity and have RNA polymerase II-cis regulatory region sequence-specific DNA binding activity. |
| *ZNF782* | Encodes for Zinc Finger Protein 782. Thought to enable DNA-binding transcription factor activity, RNA polymerase II-specific and RNA polymerase II-cis regulatory region sequence-specific DNA binding activity. |

**Supplementary Table 2.** Differentially expressed genes versus control for ROHHAD and CCHS neurons. Function was derived from Genecards.org. Safran, M., et al. (2021). The GeneCards Suite. *Practical Guide to Life Science Databases*. I. Abugessaisa and T. Kasukawa. Singapore, Springer Nature Singapore**:** 27-56.

## Supplementary Figures


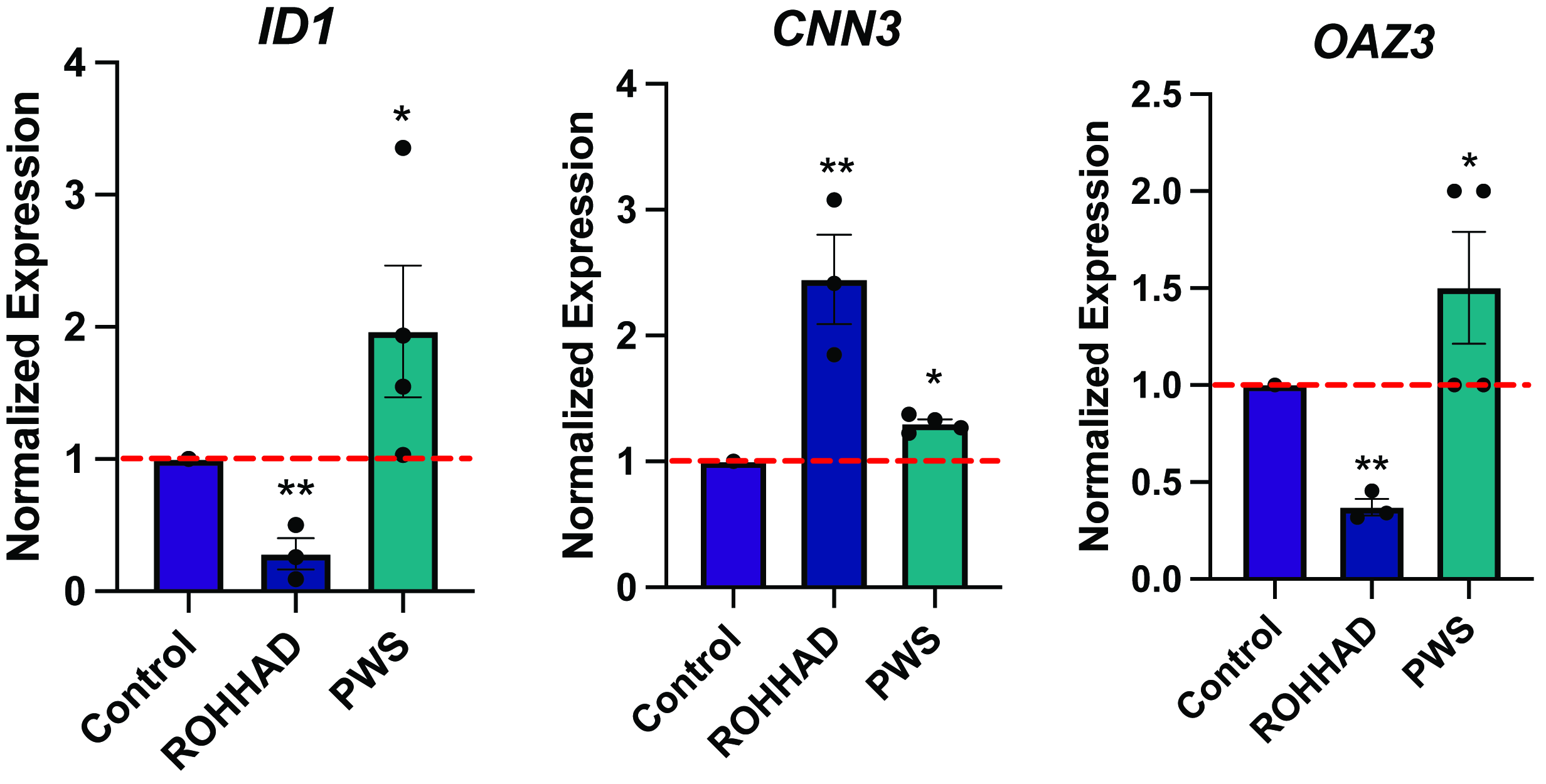


**Supplementary Figure 1. Significantly differentially expressed genes in common for ROHHAD and PWS versus control subjects.** RNAseq data from the experiment presented here and our previously published RNAseq data for PWS versus control neurons (27) were analyzed for overlapping genes unique to ROHHAD and PWS versus control neurons (*p*-value < 0.05, FDR < 0.05, fold-change <0.5 or >1.5). Bar graphs represent average RNAseq expression for each group across the three 3 these genes. Expression was normalized to the average of the control expression in each study. The dashed red line indicates control expression level. None of these genes have an obvious role in the obesity phenotype shared by PWS and ROHHAD. Significance determined during RNAseq data analysis (*p*-value ≤ 0.05 and FDR ≤ 0.05). * = *p*-value ≤ 0.05, ** = *p*-value ≤ 0.01.


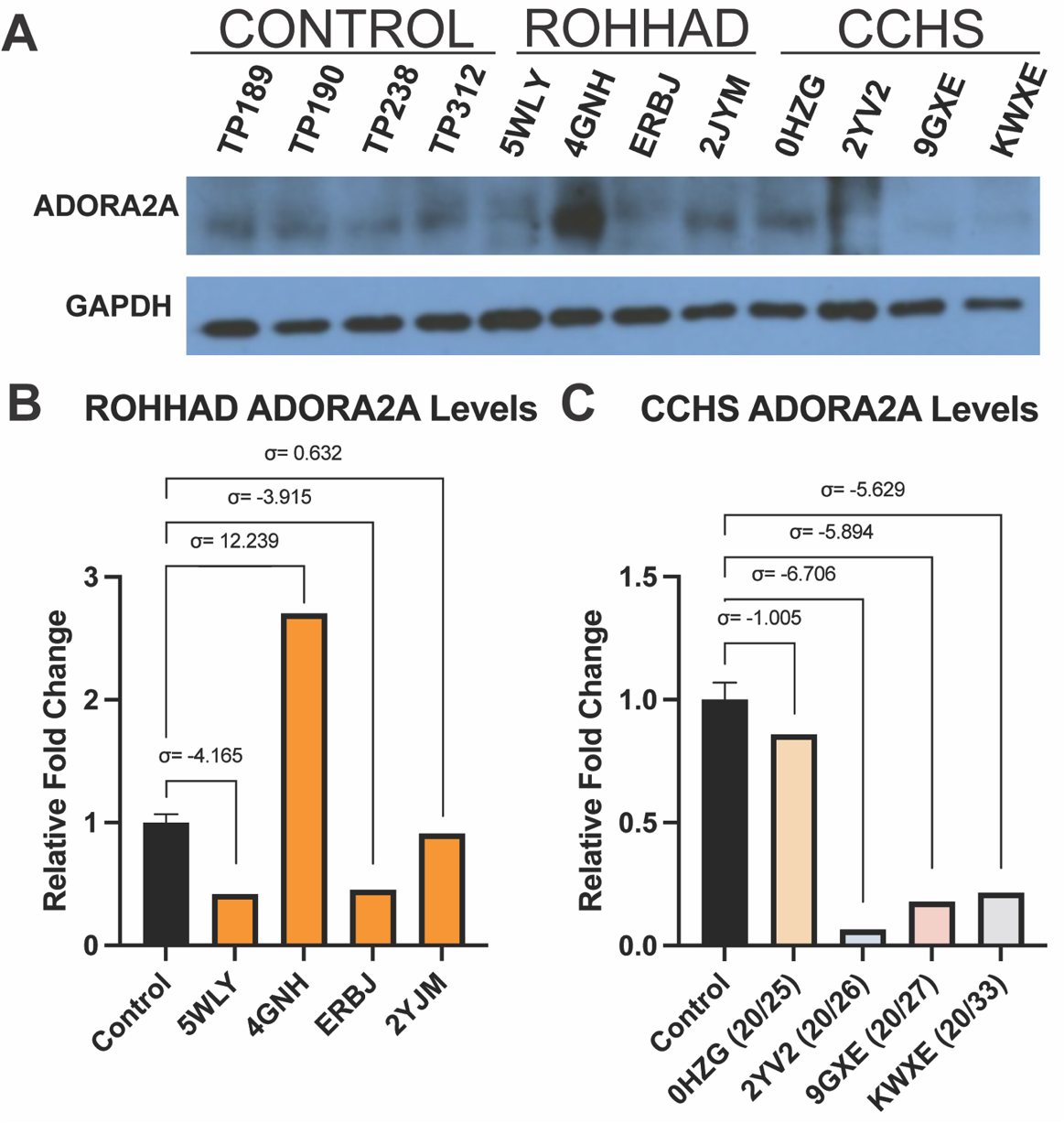


**Supplementary Figure 2. Western blot analysis of ADORA2A levels in ROHHAD and CCHS DPSC-derived neurons.** **(A)** ECL western blot of ADORA2A in Control, ROHHAD, and CCHS groups. An average of all four controls was used for normalization and comparison against each of the four individual ROHHAD and the four individual CCHS cell lines. The graphs were scaled differently for ROHHAD versus CCHS comparisons, but the average of the same 4 control samples were used in B and C. Quantification of ADORA2A protein in ROHHAD individuals, 5WLY (σ=-4.165) and ERBJ (σ=-3.915) exhibited a trend of reduction, 4GNH with an apparent increase in protein level (s=12.239), and 2JYM with a nominal increase in protein (σ=0.632). (C) ADORA2A quantification in CCHS individuals, 0HZG (20/25) shows a slight reduction (σ=-1.005), 2YV2 (20/26) exhibits a greater reduction (σ=-6.706), 9GXE (20/27) and KWXE (20/33) also show a trend in reduction (σ=-5.894, σ=-5.629 respectively). s is the standard deviation from the average of the four control samples.


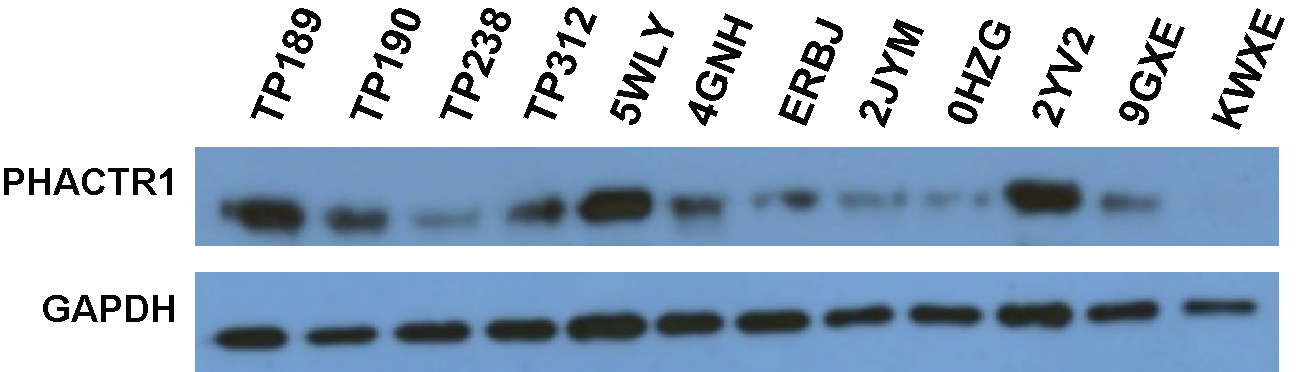


**Supplementary Figure 3. Western blot analysis of PHACTR1 levels in ROHHAD and CCHS DPSC-derived neurons.** ECL western blot of PHACTR1 in Neurotypical Control (TP189, TP190, TP238, TP312), ROHHAD (5WLY, 4GNH, ERBJ, 2JYM), and CCHS (0HZG, 2YV2, 9GXE, KWXE) subjects. There was no consistent change of PHACTR1 amongst the controls, ROHHAD, and CCHS groups, although some individual subjects did show differing expression versus the average control values.


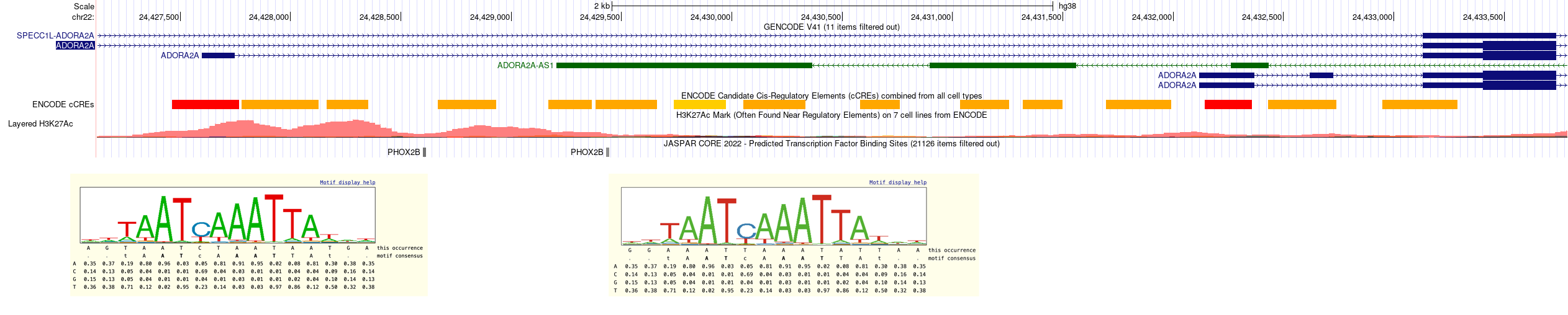


**Supplementary Figure 3. Graphic of the *ADORA2A* gene region on chr22 taken from the UCSC Genome Browser on Human (GRCh38/hg38).** The first exon of Isoform II of *ADORA2A* sits above a predicted promotor region by ENCODE cCRE (Candidate Cis-Regulatory Elements) and is in a region of open chromatin conformation as predicted by increased H3K27Ac (red bar, data from the Bernstein Lab at the Broad Institute). There are two predicted PHOX2B binding sites in this promotor region as well (70). Homology to the consensus motif for PHOX2B is shown below the predicted binding sites in the genome browser image.
